# Variations of Workload Indices Prior to Injuries: A Study in Trail Runners

**DOI:** 10.3390/ijerph17114037

**Published:** 2020-06-05

**Authors:** Sérgio Matos, Filipe Manuel Clemente, Rui Silva, José María Cancela Carral

**Affiliations:** 1 Escola Superior Desporto e Lazer, Instituto Politécnico de Viana do Castelo, Rua Escola Industrial e Comercial de Nun’Álvares, 4900-347 Viana do Castelo, Portugal; filipe.clemente5@gmail.com (F.M.C.); rui.s@ipvc.pt (R.S.); 2 Faculty of Educational Sciences and Sports Sciences, University of Vigo, 36005 Pontevedra, Spain; chemacc@uvigo.es; 3 Instituto de Telecomunicações, Delegação da Covilhã, 1049-001 Lisboa, Portugal

**Keywords:** load monitoring, fitness, performance, endurance sports

## Abstract

The purpose of this study was to compare the variations of weekly workload indices of internal and external load measures across the three weeks prior to injury occurrences in trail runners. Twenty-five trail runners (age: 36.23 ± 8.30 years old; body mass: 67.24 ± 5.97 kg; height: 172.12 ± 5.12 cm) were monitored daily for 52 weeks using global positioning systems (GPSs) to determine the total distance covered. Additionally, a rate of perceived exertion (RPE) scale was applied to determine session-RPE (sRPE: RPE multiplied by training time). The accumulated load (AL), acute: chronic workload ratio (ACWR), training monotony (TM), and training strain (TS) indices were calculated weekly for each runner. During the period of analysis, the injury occurrences were recorded. The differences were observed in AL and ACWR for sRPE and training time were significantly greater during the injury week when compared to the previous weeks. Similar evidence was found in TM and TS indices for sRPE, training time, and total distance. Furthermore, no meaningful differences were observed in AL and ACWR for total distance in the weeks prior to injury occurrence. Nevertheless, significant between-subjects variability was found, and this should be carefully considered. For that reason, an individualized analysis of the workload dynamics is recommended, avoiding greater spikes in load by aiming to keep a progressive increment of load without consequences for injury risk.

## 1. Introduction

Training is defined as the process by which athletes improve their skills through various exercises resulting from the training process [1]. During the training process, physiological and neuromuscular adaptations are expected to occur due to the load imposed on the athlete, resulting in positive and/or negative results [2]. Considering the cause–effect relationship between training and stimuli [3], monitoring the load and the athlete is one of the important mechanisms that can help coaches to maximize the positive effects of overload without exposing players to an increase in injury risk. In fact, overload is part of the training process to enhance adaptations on athletes, however, the stimuli–recovery must be considered while identification of possible risk factors (i.e., mediators of injury associated to greater spikes in load) should be tracked [4].

In recent years, load monitoring has been studied in-depth. It is a complex process, combining external factors (related to the task) and internal factors (related to the athlete), which are differentiated for each sport [4,5]. Thus, there are two types of load: external and internal [6]. External load is the physical and mechanical dimensions resulting from training planning (i.e., organization, intensity, exercise duration, etc.) [7], and internal load is the athlete’s biological response to exercise (i.e., heart rate, rate of perceived exertion, etc.) [8]. Monitoring the internal load allows one to determine an athlete’s physical and psychological responses to the imposed load (i.e., weight lifted, power output, speed, acceleration, elevation), which can be influenced by external factors such as recovery status, sleep quality, muscle soreness, stress, nutrition, and the weather [9,10]. On the other hand, monitoring external load allows creating adaptations to the athlete through variations in intensity and volume [10]. Currently, there are several valid and accurate methods for assessing loads using global positioning systems (GPSs) and accelerometers [7] to determine external load and monitor the athlete’s heart rate, blood lactate levels, and subjective perception of effort (RPE) in response to the internal load [9]. The combination of internal and external load monitoring has potential concerning the prescription and assessment of the training process and the athlete [11].

Given the relevance of monitoring workloads, several studies were developed in team [12,13] and individual sports [14,15,16] and are essential in the process of optimizing performance and preventing the risk of injury in athletes [17]. Thus, adequate training planning [18] can induce positive physiological adaptations in athletes and their performance [8]. When this does not happen, the most common effect is injury, which is pointed out as a direct consequence of training [19] and expressed by the number of training sessions/games lost [20]. Some studies examined different sports [21,22,23,24] to quantify the relationship between training and injury and showed that high workloads are associated with an increased risk of injury (i.e., overuse injuries). Clemente et al. (2019) described the training methodology applied in team sports as highly variable, both externally and internally [12]. This makes it necessary to understand whether the chronic workload to which the athlete was subjected allows “spikes” in acute workload to occur if the athlete developed the physical and psychological qualities necessary for the load to which he will be submitted [25]. Variations in load, although essential to improving performance, must be controlled considering the athlete and depending on each sport, which can lead to different adaptations in the athlete [26]. Gabbett (2020) stated that the relationship between the load and the injury must always be considered individually for each subject, as each athlete has different limits [27]. Thus, taking into account the sport and the athlete, such evidence demonstrates that monitoring allows the load to be controlled and injuries from “spikes” and wide variations to be prevented [8,28].

RPE is one of the most-used methods due to its validity and ease of application, allowing through its multiplication by the duration of the training session or competition, calculation of the session-RPE (sRPE), expressed in A.U. (arbitrary units) [29]. The sRPE reflects the general load of a training session or competition, serving as an indicator of the magnitude of the stimulus imposed on the athlete [29]. This method is often applied in team sports, such as professional soccer, in which players exhibit during the preseason (6–8 weeks before the competitions begin) an average sRPE result ranging among 321 ± 23 A.U. [30], 447 ± 209 A.U. [6], and 644 ± 224 A.U. [31]. In a study on volleyball, the preseason average for sRPE was 245.2 ± 72.3 A.U. [32]. In terms of individual sports, a four-week study on trail running by Matos et al. (2019) presented sRPE values of 213.7 ± 223.95 [14]. Similarly, with endurance runners in the preseason (three weeks), the mean sRPE was 276.4 ± 70.1 at week 1, 195 ± 37.1 at week 2, and 228.3 ± 65.2 at week 3 [15]. These results demonstrate that higher workloads (between 1500 and 2700 A.U. per week) are associated with the preseason, and the largest “spikes” occur during this time and should not increase by more than 1000 A.U. over the previous week due to being associated with a higher risk of injury in athletes [21,33,34]. 

In addition to controlling sudden increases in the load that cause the “spikes”, other factors, such as the low variability associated with high intensity/volume can be a problem [18]. In this sense, indices such as the acute: chronic workload ratio (ACWR) can be calculated from the sRPE [35]. This index makes it possible to verify whether the loads previously imposed (chronic) were sufficient for the load to be exposed (acute) [35]. ACWR values between 0.80 and 1.30 are considered as a “sweet spot” [22,36], and values greater than 1.5 are considered within the “danger zone” [25], with emphasis on values above 1.6 increasing the risk of injury by three to five times [37,38]. Regarding the training monotony and strain indices proposed by Foster (1998), monotony is calculated as the average of the workload divided by the standard deviation of the workload, and strain is the monotony multiplied by the workload [39]. Monotony and strain are used as indicators to quantify the variability of training (monotony) and the stress imposed (strain) on the athlete [40]. A study in football verified the relationships of monotony and strain with injury (trauma and illness), with monotony values of 2.59 and 2.52 and strain values of 1.01 and 1 [41]. Based on these methods, the main challenge for coaches is to understand what workloads the athlete may be subjected to, thereby enhancing performance and preventing injury [26].

It is a fact that “spikes” in the workload and high monotony increase the risk of injury. However, not all athletes are subject to these events contract injuries, suggesting that certain factors make some athletes more resistant than others, making it worthwhile to identify one’s potential injury mediators and moderators [42]. A possible mediator is mentioned by Hulin et al. (2016) among elite rugby league players in which high chronic workloads permit greater resistance to injury when followed by acute workloads of 0.85–1.35 [37]. Accordingly, the study by Malone et al. (2019) in amateur hurling players revealed that moderate workloads (between 0.9 and 1.3 ACWR and 1400 to 1900 A.U.) have a protective effect on the athlete with the injury, adding that well-developed variables such as lower body strength, faster speed, and repeated sprint are potential injury moderators for this population [43]. Another example is described in Gaelic football players—it was found that appropriate levels of aerobic capacity and extensive playing experience protect against “spikes” in the load [26]. These studies corroborate the idea presented by Windt et al. (2017) that such injury mediators and moderators function as effect modifiers [42].

Within trail running and given its short time of existence compared to the sports mentioned above, training load monitoring to improve performance only recently started to be investigated [44], with evidence on the importance of load monitoring for this population. An example of this is the study conducted by Matos et al. (2019) on trail running, which found no significant changes in the load between the four weeks studied, thus suggesting a possible lack of variation across the weeks and not following principles of progression or variability of the load aimed at promoting adaptations [14]. In addition, for trail running and in the epidemiology of injuries, several studies [45,46,47] demonstrated that the most frequent regions of injury occurrence are the lower back, hip joint, knee, ankle, and foot, while the most common injuries are of the musculoskeletal typology, such as Achilles tendinopathy, iliotibial band syndrome, femoral patellar syndrome, stress fractures, and plantar fasciitis, suggesting that overuse is the leading cause of injuries. This emphasized the importance of training periodization to avoid injuries and overtraining. The overtraining arises from a condition from overload without an adequate recovery [48]. The monitoring of the load is crucial, and it can be performed through the principles of training such as the microcycles, mesocycles, and macrocycles, thus allowing an adequate load distribution [49].

The evidence above demonstrates the importance of an adequate load distribution and monitoring training loads, facilitated through methods such as RPE, ACWR, monotony, and strain, and adequate training planning to avoid “spikes” in the workload, low variations, and exposure to the risk of injury. In addition, it is essential to understand data for all training- and athlete-related variables, which can change depending on the sport. This provides coaches and athletes with the tools needed to maximize performance and minimize the risk of injury. To the best of our knowledge, no information is reported on the relationship between workload and injury rates in trail running athletes. Thus, the purpose of this study is to compare the variations of weekly workload indices of internal and external load measures between the three weeks prior to an injury occurrence in trail runners.

## 2. Materials and Methods 

### 2.1. Participants

The sample consisted of 25 male trail running athletes (age: 36.23 ± 8.30 years old; height: 172.12 ± 5.12 cm; body mass: 67.24 ± 5.97 kg) participating in the Portuguese trail running championships during the 2018/2019 season, and with minimum ranking ITRA (International Trail Running Association) performance index of 600, with this score based on the mean of the five best race scores by a runner in the last 36 months, on a scale of 1000 points.

For 52 weeks, athletes were monitored daily using global positioning systems (GPSs) to collect data related to horizontal displacements (distance and duration), and the rate of perceived exertion (RPE) was considered to determine the session-RPE. All athletes reported data of training sessions, defined by the athlete or a trainer. The inclusion criteria were (i) participation in the national trail running championships, (ii) more than three years of experience in the sport, (iii) registration of all training sessions, and (iv) not being injured for more than three consecutive weeks in the last 12 months. Before the study began, all athletes were informed of the objectives, procedures, and protocol of the study, and they voluntarily signed an informed consent. The study was carried out following the Helsinki Declaration’s ethical recommendations for studies in humans.

### 2.2. Experimental Approach to the Problem

This study followed a cohort study design. Data from 148.12 ± 57.53 training sessions were collected for each athlete regarding the training load (training time, RPE session (sRPE), and distance covered), as well as the occurrence of injuries, their typology, and loss time. Periodically (every four months), the athletes’ anthropometric data were also recorded. In all training sessions, GPSs were used to collect data related to external load. Weekly, the acute load indices (sum of weekly training load), acute: chronic workload ratio, training monotony, and training strain were calculated for each measure (i.e., sRPE, distance covered, and session duration). Training weeks were defined as starting on Monday and ending on Sunday. Concerning the injuries recorded, a total of 38 injuries with downtimes were recorded and classified according to severity (1 to 3 days, 4 to 7 days, and 8 to 21 days). From the included injuries, 20 were in grade 1, 8 in grade 2 and 10 in grade 3 [50]. 

Considering the objective of the study and identifying the occurrence of injuries, the three weeks prior to each injury occurrence were defined, with the training load indices being compared between the three weeks prior to the injury. The weeks were classified as follows: (i) injury week (week in that the injury occurred), (ii) week-1 (week of training prior to injury week), (iii) week-2 (two weeks prior to injury week), and (iv) week-3 (three weeks prior to injury week). 

### 2.3. Injuries Occurrence

Through an online form, for all training sessions, the athletes were asked about possible injury episodes. Thus, in injury episodes, information such as the mechanism, typology, and location of the injury, as well as loss time, was collected to enable an assertive analysis of the severity of the injury and to verify the relationship between the workload indexes and the injury. The athletes were instructed on how to fill out the questionnaire, familiarized, and followed-up by a researcher who recorded the data whenever they occurred. Only musculoskeletal and sprain injuries were considered to impede athletes’ practice; in the case of dermatological injuries, these do not impede practice but only cause limitations. Thirteen athletes contracted injuries, resulting in 38 injuries and categorized according to type (five were dermatological and 33 were musculoskeletal). Of all the injuries, 25 prevented practice for 1–3 days, ten prevented practice for 4–7 days, and three prevented practice for 8–21 days.

### 2.4. Training Load Monitoring

#### 2.4.1. Distance Covered

In all training sessions, watches with GPS technology were used to collect data on horizontal displacements (distance and duration). Each athlete wore a Polar V800 watch (37 × 56 × 12.7 mm, weight: 79 g) with integrated GPS (Polar, Finland). The watch was tested for validity in previous studies and acceptable values of accuracy were verified [51]. The total distance covered per session was recorded by each athlete.

#### 2.4.2. Rate of Perceived Exertion

The rate of perceived exertion was monitored using the Borg CR-10 scale [52], which was used to collect the athletes’ subjective perceptions of effort through the question, "How hard was the training session?" with 1 corresponding to very light activity intensity and 10 representing maximal exertion [29]. This scale was introduced to the athletes two weeks before the beginning of the study so they could familiarize themselves with it, thus making their responses more precise. This procedure was performed 30 minutes after the end of each training session, ensuring that it was performed individually. In addition, the athlete provided information for any segment that influenced the classification (more difficult or easier) [29,53]. Session-RPE was determined based on the RPE values provided by the athletes and the duration of the training session (in minutes), and was expressed in arbitrary units (A.U.) [29]. This procedure was performed daily during all training sessions, as it is a valid measure for quantifying internal load [53].

#### 2.4.3. Workload Indices

Based on the sRPE values, training time, and distance covered, the following indices were calculated: (i) weekly training load (sum of all training loads for the week), (ii) acute: chronic workload ratio (ACWR: calculated by dividing the acute load (current week’s training load) by the chronic workload (average of the previous four weeks’ workload) [35], (iii) training monotony (average of workload) of the last seven days divided by the standard deviation of the last seven days’ workload) [39], and (iv) training strain (monotony multiplied by workload) [39]. In particular, the ACWR used the history of training load or players prior to each week included (to have the correct analysis considering their training load). For example, the ACWR in week-3 was calculated based on the weeks -3, -4, -5, and -6, despite weeks -4, -5, and -6 not being presented in this particular study.

## 3. Statistical Analysis

The results are presented in the form of means and standard deviations. The data were preliminarily checked for normality (*p* > 0.05) and homogeneity (*p* > 0.05). After confirming both assumptions, a repeated measures ANOVA was conducted to compare the workload indices (acute load, ACWR, training monotony, and training strain) of the three measures (sRPE, total distance, and total time) between four weeks (three weeks prior to injury and the injury week itself). Bonferroni’s post hoc test was used to analyze the pairwise variations (week vs. week analysis). These statistical procedures were performed using SPSS statistical analysis software (version 24, IBM Corporation, Armonk, NY, USA). The level of statistical significance was set at *p* < 0.05. Additionally, the standardized effect size (ES) of Cohen’s d was calculated for pairwise comparisons to determine the ES. The magnitude of ES was described based on the following thresholds: ≤0.2, from 0.3 to 0.6, from 0.6 to 1.2, from 1.2 to 2.0, and >2.0 were considered trivial, small, moderate, large, and very large, respectively. 

## 4. Results

**Figure 1:**
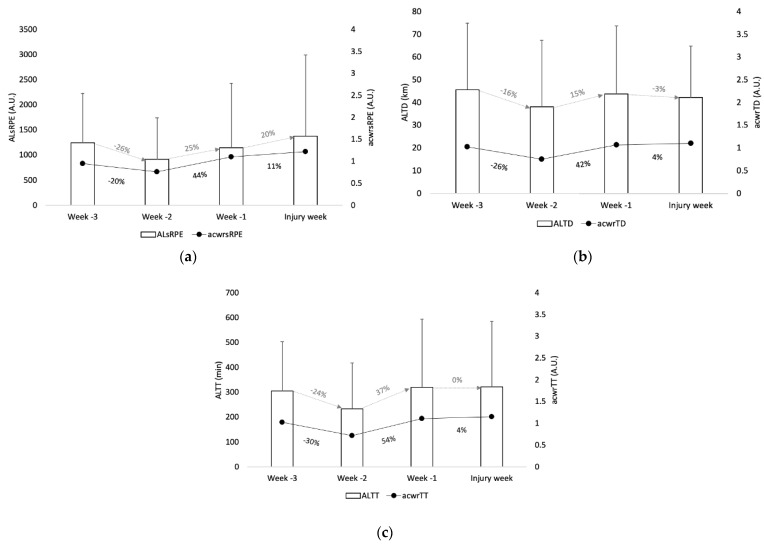
Descriptive statistics (error bar: mean and standard deviation of acute load; black circle: mean of acute: chronic workload ratio (acwr)) of (**a**) session rate of perceived exertion (sRPE), (**b**) total distance (TD), and (**c**) training time (TT) in the three weeks (w) prior injury occurrence. The grey number (%) represents the percentage of changes from one week to another for acute load and the black number (%) represents the percentage of change from one week to another in ACWR.

Descriptive statistics of acute load, acute: chronic workload ratio for the measures of sRPE, total distance (TD), and total time (TT) can be found in Figure 1. The mean of weeks -1, -2, and -3 for accumulated load sRPE (ALsRPE) was 1103 A.U. while for the injury week it was 1379 A.U. The mean of weeks -1, -2, and -3 for accumulated load total distance (ALTD) was 42 km while for the injury week it was 42 km. The mean of accumulated load training time (ALTT) for weeks -1, -2 and -3 was 287 min while for the injury week it was 321 min. Concerning the acute: chronic workload ratio (acwr), the mean of weeks -1, -2, and -3 for acwrsRPE was 0.9 A.U. while for the injury week it was 1.2 A.U. The mean of weeks -1, -2, and -3 for acwrTD was 0.9 A.U. while for the injury week it was 1.1 A.U. The mean of weeks -1, -2, and -3 for acwrTT was 1.0 A.U. while for the injury week it was 1.2 A.U.

**Figure 2:**
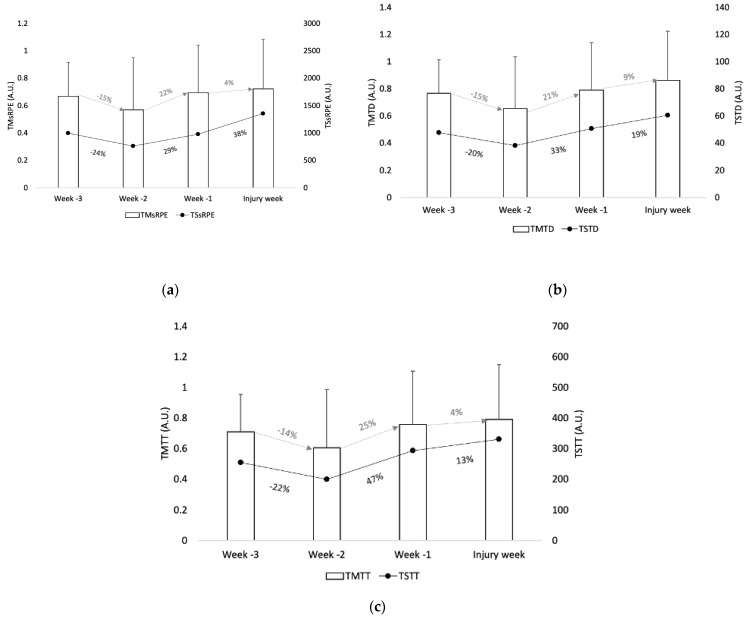
Descriptive statistics (error bar: mean and standard deviation of training monotony; black circle: mean of training strain) of (**a**) sRPE, (**b**) total distance (TD), and (**c**) training time (TT) in the three weeks (w) prior injury occurrence. The grey number (%) represents the percentage of changes from one week to another for training monotony and the black number (%) represents the percentage of change from one week to another in training strain.

Descriptive statistics of training monotony (TM) and training strain (TS) for the measures of sRPE, total distance, and total time can be found in Figure 2. The mean of weeks -1, -2, and -3 for TMsRPE was 0.6 A.U. while for the injury week it was 0.7 A.U. The mean of weeks -1, -2, and -3 for TMTD was 0.7 A.U. while for the injury week it was 0.9 A.U. The mean of TMTT for weeks -1, -2, and -3 was 0.7 A.U. while for the injury week it was 0.8 A.U. Concerning the training strain, the mean of weeks -1, -2, and -3 for TSsRPE was 913 A.U. while for the injury week it was 1359 A.U. The mean of weeks -1, -2, and -3 for TSTD was 45.6 A.U. while for the injury week it was 60.5 A.U. The mean of weeks -1, -2, and -3 for TSTT was 250 A.U. while for the injury week it was 331 A.U.

Table 1 presents the pairwise comparisons (based on the Bonferroni test and standardized effect size of Cohen) between weeks for the workload indices of acute load, acute chronic workload ratio, training monotony, and strain of total distance before injury. Repeated measures ANOVA revealed no significant differences (*p* > 0.05) for acute load, ACWR, TM, and TS workload indices of total distances between weeks.

Table 2 presents the pairwise comparisons (based on the Bonferroni test and standardized effect size of Cohen) between weeks for the workload indices of acute load, acute chronic workload ratio, training monotony, and strain of total time before injury. Repeated measures ANOVA revealed no significant differences (*p* > 0.05) for acute load, TM, and TS workload indices of training time between weeks. Significant differences between weeks were found for ACWR (*p* < 0.05).

Table 3 presents the pairwise comparisons (based on the Bonferroni test and standardized effect size of Cohen) between weeks for the workload indices of acute load, acute chronic workload ratio, training monotony, and strain of sRPE before injury. Repeated measures ANOVA revealed no significant differences (*p* > 0.05) for acute load, ACWR, TM, and TS workload indices of sRPE between weeks.

## 5. Discussion

The purpose of the present study was to compare the acute load, acute: chronic workload ratio, training monotony, and training strain of the three weeks prior to injury occurrences in trail runners. The acute load, acute: chronic workload ratio, training monotony, and training strain for external and internal load measures of total distance, training time, and sRPE were monitored and analyzed in the three weeks prior to injury occurrences in trail runners. The results showed significant weekly increases in acute load, acute: chronic workload ratio, training monotony, and training strain for sRPE, total distance, and training time in the weeks prior to injury occurrence.

Week-to-week changes in workload of more than 10–15% were related to increased injury risk [18,25]. In the present study, the results of injury week with the three weeks prior to the injury show that the values of workload indices were higher in the injury week than in the average of the three weeks prior to the injury for almost all variables (ALTD were the same value), corroborating the study by Hulin et al. [35], with results indicating that acute loads greater than chronic loads increase the risk of injury. Also, abrupt decreases in acute loads of −26%, −24% and −16% were found from week-3 to week-2, followed by abrupt increases of 25%, 37%, and 15% from week-2 to week-1 prior to injury occurrences for sRPE, training time, and total distance, respectively. For instance, workload data from 28 elite cricket players were collected in a study over a six-year period, and it was found that 57% of the injuries occurred one week after a negative training balance where the acute loads were superior to chronic loads [54]. In the present study, the same trend was observed, revealing “spikes” in workloads during the two weeks before injury occurrences, with higher acute workloads being related to chronic loads. Also, these “spikes” were preceded by abrupt decreases in workload for the external and internal measures from week-3 to week-2, considering the three-week period before injury occurrence. Given that, as mentioned above, although sudden increases or decreases in acute workload are associated with higher injury risk [35,38,55], it may be relevant to consider the significant changes in the workloads in both directions (decrease and increase, as found in the descriptive statistics of this study).

Considering the volume of acute workloads, the results showed a decrease of 16% for total distance from week-3 to week-2, followed by an increase of 15% from week-2 to -1. As for training time, a drop of 24% from week-3 to -2 was seen, followed by an increase of 37% in acute load from week-2 to week-1 prior to injury. In contrast, Nielson et al. [56] in a one-year prospective cohort study analyzed the associations between two-week sudden load distance change and injury occurrence and found that novice runners were more susceptible to contract running-related injuries only when their distance acute load was increased by more than 30% when compared to runners whose weekly distance load was increased by 10% to 29%. Also, in a randomized controlled trial over a 13-week period that compared an 8-week standard training group (~25% weekly change) with an intervention group with a ~10% progressive weekly load, no significant differences were found between the groups, revealing incidences of 20.8% for the 10% progressive load and 20.3% for the control group. The aforementioned studies suggest that the well-established 10% rule of load progression in endurance sports may be misleading coaches, as there is a lack of evidence to support its relationship with injury risk reduction [57].

In relation to the acute: chronic workload ratio, recent research in other sports such as rugby and soccer showed the importance of using this ratio in external and internal measures to prevent injuries and to monitor athletes’ preparedness for training [21,25,36]. The results of this study show that in relation to external measures, the injury week had greater acute: chronic workload ratios than week-2 for total distance and training time. The same was found for the internal measure of sRPE. Overall, in the present study, the acute: chronic workload ratios remained between 0.7 and 1.2 between the four-week periods for total distance, training time, and sRPE. 

These results are in line with the U-shape curve referred to by Gabbett [25], which suggests that when acute training loads are similar to chronic training loads (giving an acute: chronic workload ratio of between 0.8 and 1.3), the risk of injury is reduced. For instance, it was demonstrated that higher chronic workloads relative to the athlete’s fitness have a protective effect in relation to injury occurrence if the acute workloads relative to the athlete’s fatigue remain lower than the chronic loads. In one study on 53 elite rugby players over a two-season period, it was reported that players with high chronic loads are better protected against injuries when acute loads are increased. However, it was also found that when high chronic loads are combined with a very high acute: chronic workload ratio (i.e., more than approximately 1.5), athletes are less protected, exhibiting a 28.6% risk of injury [35]. Although the results in this study revealed that the acute: chronic workload ratios for sRPE, total distance, and training time remained between 0.7 and 1.3, it is necessary to analyze the data carefully, as the standard deviations are very similar to the mean values, revealing a very high coefficient of variation and suggesting high variation within subjects [58], which is especially evident in week-3 for all external and internal measures. This means that, in reality, the acute: chronic workload ratios were high to very high at the intrasubject level for some of the athletes. Also, the “sweet spot” of the acute: chronic workload ratio might not be as applicable to trail running as it is for other sports [21]. 

Regarding training monotony and training strain, the results revealed decreases in training monotony of around 15% and in training strain of 20% to 24%, followed by an increase in training monotony from 4% to 25% and in training strain from 13% to 47%, for sRPE, total distance, and training time prior to injury occurrence. Greater changes were observed for training strain from week-1 to the injury week for sRPE. The present results are in contrast with those found by Clemente et al. [12] in relation to training monotony, which showed a decrease over a ten-week monitoring period, though higher values (3.8 A.U.) were revealed in comparison to the present results (0.9 A.U.), demonstrating that although monotony indices higher than 2 A.U. represent a risk factor for illness and overtraining in players [33], the results showed that lower monotony values (0.9 A.U.) can also result in injury since such occurrence is multifactorial. However, the same authors found a progressive increase in training strain, reaching 1751 A.U., which is higher than the maximum value found in the present study (1359 A.U.). In a study of 130 elite soccer players over one season, Delecroix et al. [40] found that increases in training strain values were more closely related to injury risk than increases in training monotony, also showing that the increases in training monotony may be a factor of injury protection [39]. Increases in training strain are expected when a high monotonous and homogeneous weekly training loads are observed [59]. 

The main finding of the present study is that in the three weeks before an injury occurred, sudden weekly changes occurred in acute loads for sRPE, training time, and total distance. Training strain and training monotony after an abrupt decrease in the first week were followed by progressive increases until the injury week. Another finding was related to the masked “sweet spot” of acute: chronic workload ratios revealing high within-subjects variation with high ratios for some of the athletes. To the best of our knowledge, this study was the first to analyze the relationships of acute load, acute: chronic workload ratio, training monotony, and training strain for the three weeks prior to injuries in trail running athletes.

This study has some limitations. The size of the sample is one of the main limitations, opting only for the use of males due to the lack of commitment of females to participate in the study and consequently the number of females achieved would not allow a comparison between genders. Another limitation is that the population analyzed is nonprofessional, thus the training process is adjusted with the possibilities and knowledge of these athletes. Another issue is related to the familiarization of these kinds of athletes with the sRPE, which could have caused them to underrate or overrate their effort. The use of additional accelerometry-based data (i.e., accelerations, mechanical work) would be of paramount interest in future studies. An identification of uphill and downhill magnitudes as external load must be also considered in future studies, mainly regarding the specificity of the trail running. Finally, an intraweek analysis could be interesting to detect possible abrupt changes within-week that were not possible observe in the present study. An additional comparison between macrocycles without injury with microcycle with injuries must be conducted in the future.

## 6. Conclusions

There were significant weekly increases in acute load, acute: chronic workload ratio, training monotony, and training strain for sRPE, total distance, and training time in the weeks prior to an injury’s occurrence. The variations in the weekly load demonstrate a drop in the workloads for the external and internal measures from week-3 to week-2, which was followed by increases from week-2 until the injury week. A similar trend was observed for training strain and training monotony—after an abrupt fall in the first week, progressive increases were recorded until the injury week. The values obtained for the acute: chronic workload ratios for total distance, training time, and sRPE ranged from 0.7 to 1.3 A.U.; these values are considered as being within the “sweet spot,” however, and due to the high intrasubject variation with high proportions for some athletes (large SD), the acute: chronic workload ratios values in the case of trail runners may be masked, suggesting the “sweet spot” for trail runners may be different.

These results suggest that athletes and coaches should pay special attention to the periodization of training and the monitoring of workloads, avoiding high monotony, and allowing changes in loads that induce adaptation to the stimulus. Therefore, the acute loads must increase progressively and consider both chronic load and moderator variables to prevent significant increases in the injury risk.

## Figures and Tables

**Table 1:** Between-weeks variations of workload indices based on total distance before injury.

Index	Week-3 Mean ± SD	Week-2 Mean ± SD	Week-1 Mean ± SD	Injury Week Mean ± SD	*p* Pairwise Comparisons	Effect Size (d) | Magnitude Pairwise Comparisons
AL (km)	45.6 ± 29.4	38.1 ± 29.3	43.7 ± 30.1	42.3 ± 22.6	w-3 vs. w-2: 0.205 w-3 vs. w-1: 0.828 w-3 vs. Iw: 0.651 w-2 vs. w-1: 0.458 w-2 vs. Iw: 0.460 w-1 vs. Iw: 0.843	w-3 vs. w-2: 0.256, small w-3 vs. w-1: 0.064, trivial w-3 vs. Iw: 0.126, trivial w-2 vs. w-1: −0.189, trivial w-2 vs. Iw: −0.161, trivial w-1 vs. Iw: 0.053 trivial
ACWR (A.U.)	1.0 ± 0.9	0.7 ± 0.5	1.1 ± 0.7	1.1 ± 0.6	w-3 vs. w-2: 0.229 w-3 vs. w-1: 0.857 w-3 vs. Iw: 0.681 w-2 vs. w-1: 0.102 w-2 vs. Iw: 0.077 w-1 vs. Iw: 0.792	w-3 vs. w-2: 0.412 small w-3 vs. w-1: −0.124 trivial w-3 vs. Iw: −0.131 trivial w-2 vs. w-1: −0.658 moderate w-2 vs. Iw: −0.724 moderate w-1 vs. Iw: <0.001 trivial
TM (A.U.)	0.8 ± 0.3	0.7 ± 0.4	0.8 ± 0.4	0.9 ± 0.5	w-3 vs. w-2: 0.217 w-3 vs. w-1: 0.832 w-3 vs. Iw: 0.406 w-2 vs. w-1: 0.236 w-2 vs. Iw: 0.063 w-1 vs. Iw: 0.561	w-3 vs. w-2: 0.283 small w-3 vs. w-1: <0.001 trivial w-3 vs. Iw: −0.243 small w-2 vs. w-1: −0.250 small w-2 vs. Iw: −0.442 small w-1 vs. Iw: −0.221 small
TS (A.U.)	47.8 ± 43.3	38.2 ± 38.7	50.7 ± 50.7	60.5 ± 99.9	w-3 vs. w-2: 0.269 w-3 vs. w-1: 0.825 w-3 vs. Iw: 0.487 w-2 vs. w-1: 0.289 w-2 vs. Iw: 0.279 w-1 vs. Iw: 0.613	w-3 vs. w-2: 0.234 small w-3 vs. w-1: −0.062 trivial w-3 vs. Iw: −0.165 trivial w-2 vs. w-1: −0.277 small w-2 vs. Iw: −0.294 small w-1 vs. Iw: −0.124 trivial

AL: acute load; ACWR: acute: chronic workload ratio; TM: training monotony; TS: training strain; w-3: three weeks before the injury week; w-2: two weeks before the injury week; w-1: one week before the injury week; Iw: injury week; km: kilometers; A.U.: arbitrary units; *p*: *p*-value corresponding to the Bonferroni test; d: standardized effect size of Cohen corresponding to pairwise comparisons; SD: standard deviation.

**Table 2:** Between-weeks variations of workload indices based on training time before injury.

Index	Week-3 Mean ± SD	Week-2 Mean ± SD	Week-1 Mean ± SD	Injury Week Mean ± SD	*p* Pairwise Comparisons	Effect Size (d) | Magnitude Pairwise Comparisons
AL (min)	305.7 ± 198.4	233.4 ± 184.8	320.7 ± 273.5	321.1 ± 263.9	w-3 vs. w-2: 0.074 w-3 vs. w-1: 0.793 w-3 vs. Iw: 0.759 w-2 vs. w-1: 0.145 w-2 vs. Iw: 0.126 w-1 vs. Iw: 0.993	w-3 vs. w-2: 0.377 small w-3 vs. w-1: −0.063 trivial w-3 vs. Iw: −0.066 trivial w-2 vs. w-1: −0.374 small w-2 vs. Iw: −0.385 small w-1 vs. Iw: −0.002 trivial
ACWR (A.U.)	1.0 ± 0.9	0.7 ± 0.5	1.1 ± 0.8	1.2 ± 0.6	w-3 vs. w-2: 0.192 w-3 vs. w-1: 0.686 w-3 vs. Iw: 0.579 w-2 vs. w-1: 0.054 w-2 vs. Iw: 0.047 w-1 vs. Iw: 0.893	w-3 vs. w-2: 0.412 small w-3 vs. w-1: −0.117 trivial w-3 vs. Iw: −0.262 small w-2 vs. w-1: −0.600 moderate w-2 vs. Iw: −0.905 small w-1 vs. Iw: −0.141 trivial
TM (A.U.)	0.7 ± 0.3	0.6 ± 0.4	0.8 ± 0.4	0.8 ± 0.4	w-3 vs. w-2: 0.222 w-3 vs. w-1: 0.648 w-3 vs. Iw: 0.395 w-2 vs. w-1: 0.164 w-2 vs. Iw: 0.058 w-1 vs. Iw: 0.748	w-3 vs. w-2: 0.283 small w-3 vs. w-1: −0.283 small w-3 vs. Iw: −0.283 small w-2 vs. w-1: −0.500 small w-2 vs. Iw: −0.500 small w-1 vs. Iw: 0.000 trivial
TS (A.U.)	256.2 ± 216.0	200.0 ± 206.1	294.0 ± 294.4	331.2 ± 503.6	w-3 vs. w-2: 0.239 w-3 vs. w-1: 0.591 w-3 vs. Iw: 0.396 w-2 vs. w-1: 0.152 w-2 vs. Iw: 0.218 w-1 vs. Iw: 0.704	w-3 vs. w-2: 0.266 small w-3 vs. w-1: −0.146 trivial w-3 vs. Iw: −0.194 trivial w-2 vs. w-1: −0.370 small w-2 vs. Iw: −0.341 small w-1 vs. Iw: −0.090 trivial

AL: acute load; ACWR: acute: chronic workload ratio; TM: training monotony; TS: training strain; w-3: three weeks before the injury week; w-2: two weeks before the injury week; w-1: one week before the injury week; Iw: injury week; min: minutes; A.U.: arbitrary units; *p*: *p*-value corresponding to Bonferroni test; d: standardized effect size of Cohen corresponding to pairwise comparisons.

**Table 3:** Between-weeks variations of workload indices based on sRPE (session-rated of perceived exertion) before injury.

Index	Week-3 Mean±SD	Week-2 Mean±SD	Week-1 Mean±SD	Injury Week Mean±SD	*p* Pairwise Comparisons	Effect Size (d) | Magnitude Pairwise Comparisons
AL (A.U.)	1244.0 ± 982.3	917.1 ± 827.3	1147.9 ± 1282.0	1378.8 ± 1617.0	w-3 vs. w-2: 0.047 w-3 vs. w-1: 0.717 w-3 vs. Iw: 0.618 w-2 vs. w-1: 0.397 w-2 vs. Iw: 0.115 w-1 vs. Iw: 0.356	w-3 vs. w-2: 0.360 small w-3 vs. w-1: 0.084 trivial w-3 vs. Iw: −0.101 trivial w-2 vs. w-1: −0.214 small w-2 vs. Iw: −0.360 small w-1 vs. Iw: −0.158 trivial
ACWR (A.U.)	0.9 ± 0.9	0.8 ± 0.7	1.1 ± 0.9	1.2 ± 0.6	w-3 vs. w-2: 0.493 w-3 vs. w-1: 0.545 w-3 vs. Iw: 0.277 w-2 vs. w-1: 0.134 w-2 vs. Iw: 0.052 w-1 vs. Iw: 0.655	w-3 vs. w-2: 0.124 trivial w-3 vs. w-1: −0.222 small w-3 vs. Iw: −0.392 small w-2 vs. w-1: −0.372 small w-2 vs. Iw: −0.614 moderate w-1 vs. Iw: −0.131 trivial
TM (A.U.)	0.7 ± 0.2	0.6 ± 0.4	0.7 ± 0.3	0.7 ± 0.4	w-3 vs. w-2: 0.217 w-3 vs. w-1: 0.790 w-3 vs. Iw: 0.536 w-2 vs. w-1: 0.202 w-2 vs. Iw: 0.083 w-1 vs. Iw: 0.712	w-3 vs. w-2: 0.316 small w-3 vs. w-1: <0.001 trivial w-3 vs. Iw: <0.001 trivial w-2 vs. w-1: −0.283 small w-2 vs. Iw: −0.250 small w-1 vs. Iw: 0.000 trivial
TS (A.U.)	997.4 ± 1021.9	760.8 ± 882.7	981.3 ± 1168.7	1359.0 ± 2538.9	w-3 vs. w-2: 0.184 w-3 vs. w-1: 0.949 w-3 vs. Iw: 0.379 w-2 vs. w-1: 0.401 w-2 vs. Iw: 0.214 w-1 vs. Iw: 0.396	w-3 vs. w-2: 0.248 small w-3 vs. w-1: 0.015 trivial w-3 vs. Iw: −0.187 small w-2 vs. w-1: −0.213 small w-2 vs. Iw: −0.315 small w-1 vs. Iw: −0.191 trivial

AL: acute load; ACWR: acute: chronic workload ratio; TM: training monotony; TS: training strain; w-3: three weeks before the injury week; w-2: two weeks before the injury week; w-1: one week before the injury week; Iw: injury week; A.U.: arbitrary units; *p*: *p*-value corresponding to the Bonferroni test; d: standardized effect size of Cohen corresponding to pairwise comparisons.
